# Increased Amplitude of the P3a ERP Component as a Neurocognitive Marker for Differentiating Amnestic Subtypes of Mild Cognitive Impairment

**DOI:** 10.3389/fnagi.2018.00019

**Published:** 2018-02-12

**Authors:** Kenia S. Correa-Jaraba, Mónica Lindín, Fernando Díaz

**Affiliations:** Laboratorio de Psicofisioloxía e Neurociencia Cognitiva, Facultade de Psicoloxía, Universidade de Santiago de Compostela, Galicia, Spain

**Keywords:** amnestic mild cognitive impairment (aMCI), Alzheimer’s disease (AD), biomarkers, event-related potentials (ERPs), P3a, involuntary attention

## Abstract

The event-related potential (ERP) technique has been shown to be useful for evaluating changes in brain electrical activity associated with different cognitive processes, particularly in Alzheimer’s disease (AD). Longitudinal studies have shown that a high proportion of people with amnestic mild cognitive impairment (aMCI) go on to develop AD. aMCI is divided into two subtypes according to the presence of memory impairment only (single-domain aMCI: sdaMCI) or impairment of memory and other cognitive domains (multi-domain aMCI: mdaMCI). The main aim of this study was to examine the effects of sdaMCI and mdaMCI on the P3a ERP component associated with the involuntary orientation of attention toward unattended infrequent novel auditory stimuli. Participants performed an auditory-visual distraction-attention task, in which they were asked to ignore the auditory stimuli (standard, deviant, and novel) and to attend to the visual stimuli (responding to some of them: Go stimuli). P3a was identified in the Novel *minus* Standard difference waveforms, and reaction times (RTs) and hits (in response to Go stimuli) were also analyzed. Participants were classified into three groups: Control, 20 adults (mean age (M): 65.8 years); sdaMCI, 19 adults (M: 67 years); and mdaMCI, 11 adults (M: 71 years). In all groups, the RTs were significantly longer when Go stimuli were preceded by novel (relative to standard) auditory stimuli, suggesting a distraction effect triggered by novel stimuli; mdaMCI participants made significantly fewer hits than control and sdaMCI participants. P3a comprised two consecutive phases in all groups: early-P3a (e-P3a), which may reflect the orienting response toward the irrelevant stimuli, and late-P3a (l-P3a), which may be a correlate of subsequent evaluation of these stimuli. The e-P3a amplitude was significantly larger in mdaMCI than in sdaMCI participants, and the l-P3a amplitude was significantly larger in mdaMCI than in sdaMCI and Control participants, indicating greater involuntary capture of attention to unattended novel auditory stimuli and allocation of more attentional resources for the subsequent evaluation of these stimuli in mdaMCI participants. The e-P3a and l-P3a components showed moderate to high sensitivity and specificity for distinguishing between groups, suggesting that both may represent optimal neurocognitive markers for differentiating aMCI subtypes.

## Introduction

Epidemiological and sociodemographic evidence indicates an increasing prevalence of Alzheimer’s disease (AD) associated with the continuous relative increase in the aging population worldwide (Fratiglioni et al., 2000; Bickel, 2001; Juckel et al., 2008; Rocca et al., 2012). AD has a profound effect on the quality of life of the sufferers, their families and carers, as well as a strong economic impact (Vellone et al., 2008; Sosa-Ortiz et al., 2012; Wimo et al., 2013). Accurate and early diagnosis of AD is a major public health concern, as although AD cannot yet be halted or reversed, recent dementia-specific pharmacological advances can slow progression of the disease (Vecchio and Määttä, 2011).

People diagnosed with amnestic mild cognitive impairment (aMCI) display symptoms that indicate possible progression to AD but that do not interfere with daily living (Petersen, 2004; Fisk, 2005; Petersen et al., 2014). Such people frequently show signs of memory impairment only (single-domain aMCI: sdaMCI) or impairment of memory and other cognitive domains (multi-domain aMCI: mdaMCI). It has been suggested that both subtypes represent part of a continuous spectrum of MCI, with mdaMCI being the most severe form of aMCI in terms of prognosis (Fischer et al., 2007; Petersen and Negash, 2008).

The identification of biomarkers of aMCI could help clinicians make objective diagnoses, thus enabling the early application of therapies with the aim of slowing down progression to AD and also providing opportunities for preventing the disease via population screening (Henry et al., 2013). Identification of biomarkers of aMCI usually requires the application of invasive (e.g., analysis of cerebrospinal fluid) and/or expensive methods [e.g., positron emission tomography (PET) or functional magnetic resonance image (fMRI)] (Small et al., 2006; Hämäläinen et al., 2007; Perneczky et al., 2011). However, the event-related potential (ERP) technique is non-invasive, simple and inexpensive (Rossini et al., 2006), making it an ideal candidate method for identifying biomarkers (Thies et al., 1999; Jackson and Snyder, 2008).

The ERP technique is well suited to detecting and quantifying changes in brain electrical activity associated with cognitive deficits (Katada et al., 2004). In fact, some ERP components have been found to be altered at early stages of AD (Golob et al., 2009; Quiroz et al., 2011) and in MCI (see Vecchio and Määttä, 2011; Bidelman et al., 2017). In addition, changes in ERP components have been shown to be potentially useful as biomarkers of the progress of cognitive impairment and subsequent conversion to dementia in individuals with MCI (for a review, see Cecchi et al., 2015).

Some ERP studies have used the novelty oddball paradigm with the aim of evaluating the brain electrical activity related to orienting response. In this paradigm, three types of stimuli are presented: standard, target, and novel. The P3b ERP component, which reflects attentional and memory processes, is elicited in response to target stimuli. In turn, unexpected stimuli (novel or deviant) elicit a frontocentral component, namely P3a (or novelty-P3; Courchesne et al., 1975; Squires et al., 1975; Escera et al., 2000), considered a psychophysiological index of the orienting response (Squires et al., 1975; Cycowicz and Friedman, 1997; Friedman et al., 2001). P3a has a shorter peak latency and a more frontal topographical distribution than P3b and is also evoked in different conditions (Squires et al., 1975; Escera et al., 2000).

The P3a component is identified at approximately 300 ms from deviation onset (Friedman et al., 2001; Horváth et al., 2009). Generation of P3a seems to involve a broad network of cortical regions, including the prefrontal cortex, cingulate gyrus, and hippocampus (Knight et al., 1989; Baudena et al., 1995; Halgren et al., 1995; Alho et al., 1998). Different studies suggest that P3a may represent evidence for “transient activation in the neural network involved in a variety of cognitive tasks that demand continual updating of task-set information for selection of goal-directed actions” (Barcelo et al., 2006; Escera and Corral, 2007; as cited in Correa-Jaraba et al., 2016). It has also been proposed that rather than reflecting the switch itself, the P3a component may also indicate the initial unhitching of the focus of attention from the current information with the aim of preparing for switching attention (Berti, 2008; as cited in Correa-Jaraba et al., 2016).

It has also been proposed that P3a is elicited in two phases in response to deviant (Yago et al., 2001; Correa-Jaraba et al., 2016) and novel sounds in young participants (Escera et al., 1998, 2001; Correa-Jaraba et al., 2016) and in middle-aged and old adults (Correa-Jaraba et al., 2016). Mager et al. (2005) observed an early P3a phase and a consecutive P3a phase (about of 330 ms) in response to novel auditory stimuli, in young and middle-aged adults; nevertheless, the latter phase could not be identified in all participants and was not evaluated (as cited in Correa-Jaraba et al., 2016). In young, middle-aged and old adults, Correa-Jaraba et al. (2016) distinguished an early phase, denominated early-P3a (e-P3a), and a later phase, named late-P3a (l-P3a), which were considered indexes of the orienting of attention toward - and evaluation of - the infrequent unexpected (novel or deviant) stimuli, respectively (for a review see Correa-Jaraba et al., 2016).

The P3a component is affected in several psychiatric and neurological disorders (Kaipio et al., 1999, 2000; Polo et al., 2003; Lepistö et al., 2004; Gumenyuk, 2005); however, the findings of the few studies concerning P3a in AD have been to some extent inconsistent. Thus, some authors observed a longer P3a latency in AD patients than in healthy controls, suggesting delayed orientation to the deviant stimuli in the former (Frodl et al., 2002; Juckel et al., 2008). On the other hand, other authors observed that the P3a amplitude was smaller in people with mild AD than in healthy controls (Cecchi et al., 2015), a finding that is in accordance with the decline in attention and executive function observed in people with mild AD during neuropsychological testing (Baudic et al., 2006; Cecchi et al., 2015). However, yet other authors did not observe any difference in the P3a component parameters between AD patients and controls (Yamaguchi et al., 2000).

If AD patients show changes in P3a parameters relative to controls, it is possible that similar changes can be observed in people with aMCI. However, we are not aware of the existence of published studies evaluating P3a parameters in people with aMCI.

The current study was hence designed to evaluate the effects of aMCI on the P3a component, recorded in response to irrelevant novel auditory stimuli. The specific aims were as follows: (1) to identify and characterize the two phases of P3a (e-P3a and l-P3a) in sdaMCI, mdaMCI and Control groups, (2) to evaluate differences between Control, sdaMCI and mdaMCI adults in the e-P3a and l-P3a parameters considered (amplitudes and latencies), and (3) to determine whether changes in these ERP parameters are sensitive and specific biomarkers of sdaMCI and mdaMCI.

An auditory-visual distraction-attention task in which the participants had to ignore the auditory stimuli (standard, deviant, and novel) and attend to the visual stimuli (responding to some of them: Go stimuli) was used. The P3a component was identified in the novel *minus* standard (N-S) difference waveforms. Reaction times (RTs) and hits to Go visual stimuli were also evaluated with the aim of studying whether both aMCI subtypes modulate the effects of involuntary capture of attention provoked by novel stimuli on both measures.

## Materials and Methods

### Sample

Fifty adults participated voluntarily in the present study and were selected from a larger sample referred to our research group from Primary Care Health Centres in Santiago de Compostela, Galicia (Spain). The participants were divided into three groups: (1) Control, comprising 20 adults (mean age: 65.8 years) with normal cognitive functioning; (2) sdaMCI, comprising 19 adults (mean age: 67 years); and (3) mdaMCI, comprising 11 adults (mean age: 71 years). The three groups were matched regarding age and years of education. The demographic and neuropsychological measures of the three groups, and the differences between groups (as determined by the corresponding analysis) are shown in **Table 1**.

**Table 1:** Mean values and standard deviation (SD, in brackets) of the demographic and neuropsychological measures.

	Control *N* = 20	sdaMCI *N* = 19	mdaMCI *N* = 11	*p* = ^∗^	Bonferroni’s^†^
Age	65.8 (7.0)	67.0 (10.0)	71.0 (6.4)	0.241	
Years of education	9.6 (4.6)	9.5 (4.4)	10.8 (5.3)	0.715	
Gender (Female/Male)	15/5	9/10	8/3		
WAIS, vocabulary	52.7 (8.7)	47.6 (12.2)	43.8 (17.0)	0.149	
MMSE	28.7 (0.9)	27.0 (1.7)	23.5 (1.7)	0.001	Control > sdaMCI > mdaMCI
CVLT (Short delay free recall)	10.3 (1.7)	4.3 (1.7)	3.1 (1.8)	0.001	Control > sdaMCI, mdaMCI
CVLT (Short-delay cued recall)	12.3 (1.7)	5.8 (2.2)	5.6 (2.1)	0.001	Control > sdaMCI, mdaMCI
CVLT (Long-delay free recall)	11.5 (1.9)	5.5 (2.7)	3.2 (2.7)	0.001	Control > sdaMCI > mdaMCI
CVLT (Long-delay cued recall)	12.5 (2.2)	6.3 (2.8)	5.2 (2.9)	0.001	Control > sdaMCI, mdaMCI
CAMCOG-R (Orientation)	9.8 (0.2)	9.4 (0.2)	8.5 (3)	0.001	Control, sdaMCI > mdaMCI
CAMCOG-R (Language)	26.3 (1.9)	25.5 (2.5)	23.2 (2.9)	0.004	Control, sdaMCI > mdaMCI
CAMCOG-R (Attention and Calculation)	7.4 (1.6)	7.3 (1.7)	5.2 (2.8)	0.008	Control, sdaMCI > mdaMCI
CAMCOG-R (Praxis)	11.3 (1.0)	10.4 (2.5)	9.2 (2.9)	0.036	Control > mdaMCI
CAMCOG-R (Perception)	6.9 (1.5)	6.3 (1.3)	6.6 (1.6)	0.521	
CAMCOG-R (Executive function)	19.8 (4.4)	16.2 (3.1)	13.5 (4.5)	0.001	Control > mdaMCI

*^∗^ANOVA (Group); ^†^p < 0.05. WAIS: Wechsler Adult Intelligence Scale; MMSE: Mini-Mental State Examination; CVLT: California Verbal Learning Test, CAMCOG-R: Cambridge Cognitive Examination.*

The present study complied with the ethical standards established in the 1964 Declaration of Helsinki (Lynöe et al., 1991) and was approved by the Galician Clinical Research Ethics Committee (Xunta de Galicia, Spain). All participants gave their written informed consent to participate in the study, after the procedures were fully explained to them. Participants did not report any medical or psychiatric diseases, history of clinical stroke, traumatic brain injury, motor-sensory deficits or substance abuse/dependence (alcohol or drug). Adults with scores higher than 10 in depression screening (Geriatric Depression Scale, GDS; Yesavage et al., 1983) were excluded. All participants were right-handed, as evaluated by the Edinburgh inventory (Oldfield, 1971), and all had normal audition and normal or corrected-to-normal vision.

All participants then underwent the following neuropsychological tests: (1) the Spanish version of the Mini-Mental State Examination (MMSE; Folstein et al., 1975; Spanish version by Lobo et al., 1999); (2) the Spanish version of the Californian Verbal Learning Test (CVLT; Delis et al., 1987; Spanish version, TAVEC by Benedet and Alexandre, 1998), which evaluates short-delay and long-delay free recall, as well as short-delay and long-delay recall with semantic cues; and (3) the Spanish version of the Cambridge Cognitive Examination (CAMCOG-R), which assesses impairments in perception, attention-calculation, praxis, language and executive functioning (Huppert et al., 1996).

When all participants were correctly diagnosed and classified as Control, sdaMCI or mdaMCI, they voluntarily attended our laboratory for psychophysiological evaluation by the ERP technique. Participants categorized as having sdaMCI or mdaMCI met the criteria for MCI outlined by Albert et al. (2011) and the criteria for aMCI proposed by Petersen (2004). All aMCI adults were characterized by self-reported memory complaints corroborated by an informant, performance of less than 1.5 SDs below age norms for the CVLT, essentially preserved activities of daily living, and no dementia. In addition, participants with mdaMCI scored less than 1.5 SDs below age- and education-related norms in the MMSE and at least two cognitive subscales of the Spanish version of the CAMCOG-R.

All control adults scored higher than the cut-off on memory, general cognitive functioning, and specific cognitive domain tests. For a more detailed description of the global samples, the criteria for inclusion/exclusion, the tests used, and the diagnosis and classification criteria, see Juncos-Rabadán et al. (2013).

### Task

**FIGURE 1:**
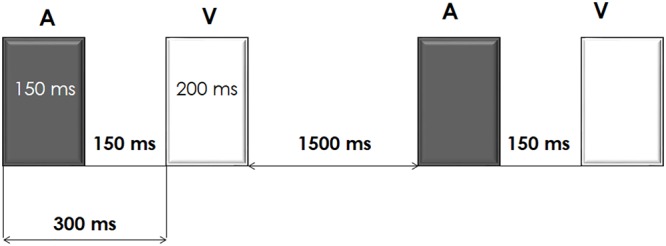
Schematic representation of the auditory-visual distraction-attention task.

Participants performed an auditory-visual distraction-attention task, which was an adaptation of the task used by Escera et al. (1998, 2001). Five hundred auditory-visual (A-V) pairs of stimuli (divided in two blocks) were presented. In each A-V pair, the duration of the auditory stimulus was 150 ms and the duration of the visual stimulus was 200 ms (onset-to-onset interval: 300 ms). An onset-to-onset interval between pairs of stimuli of 2-s duration was used. Participants were instructed to pay attention to the visual stimuli and to ignore the auditory stimuli. See diagram of the task procedure in **Figure 1**.

The unattended auditory stimuli, of intensity 75 dB SPL, were presented binaurally, via headphones. Three types of auditory stimuli were presented: a standard tone of 1000 Hz (presentation probability of 70%), a deviant tone of 2000 Hz (presentation probability of 15%), and novel stimuli (different each time, e.g., glass crashing, phone ringing; presentation probability of 15%). Three types of attended visual stimuli were also presented: numbers (2, 4, 6, and 8), with a presentation probability of 33%, letters (a, c, e, and u), with a presentation probability of 33%, and triangles (pointing right, left, upward or downward), with a presentation probability of 34%. Participants were instructed to respond (Go condition) to the numbers and to the letters by pressing two different buttons, each with a different hand (the buttons were counterbalanced among participants) and to inhibit their responses to triangles (NoGo condition).

### Electroencephalographic Recording

Recording sessions were conducted in a Faraday chamber, under attenuated levels of light and noise. The participants were seated on a comfortable chair during the electroencephalographic (EEG) recording and they were instructed to move as little as possible during the session.

Visual stimuli were presented with a subtended visual angle of 1.7 × 3.3° of arc, on a 19″ flat screen monitor (located at a distance of one meter from the participant), with a vertical refresh rate of 120 Hz. Forty-nine ring electrodes (placed in an Easycap GmbH, according to the International 10–10 system) were used for the EEG recording. An electrode placed at the tip of the nose served as the reference for all active electrodes, and an electrode at Fpz served as ground. Two electrodes placed at the outer canthi of both eyes were used to record the horizontal electro-oculogram (HEOG), whereas two electrodes placed near the right eye (one supra-orbitally and other infra-orbitally) were used to record the vertical electro-oculogram (VEOG). The EEG signal was passed through a 0.01–100 Hz analog bandpass filter and sampled at 500 Hz. The impedances were below 10 kΩ.

Off-line, ocular artifacts were corrected (using the method Gratton et al., 1983) and the EEG epochs of -150–1300 ms synchronized with each auditory stimulus (standard, deviant, or novel) were extracted (epochs in **Figures 3, 4, 5A** were shortened from -150 to 800 ms). Finally, three conditions were evaluated: Standard (standard auditory stimulus-Go visual stimulus), Deviant (deviant auditory stimulus-Go visual stimulus), and Novel (novel auditory stimulus-Go visual stimulus). At least 50 artifact-free epochs were obtained for each condition. Epochs related to the auditory stimuli-NoGo visual stimuli were not evaluated.

The ERP waveforms obtained for the three groups (Control, sdaMCI, and mdaMCI) in each condition are shown in **Figure 3**, and the ERP waveforms obtained for the three conditions (Standard, Deviant, and Novel) in each group are shown in **Figure 4**.

Prior to averaging, the signal was filtered using a 0.1–30 Hz (24 dB/octave slope) digital bandpass filter. In addition, the epochs were corrected to the mean voltage of the 150-ms pre-stimulus recording period, and those with signals exceeding ±100 μV, as well as the first five epochs of each block, were rejected.

Finally, N-S difference traces were obtained (see **Figure 5A**) with the aim of identifying and measuring P3a.

### Data Analysis

**FIGURE 2:**
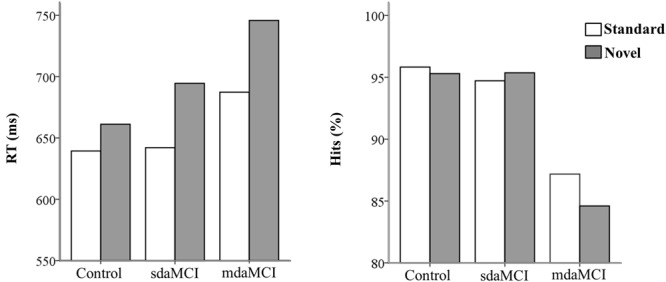
Mean values of reaction times (RT, in ms- left figure) and percentage of hits (right figure) in each condition (Novel and Standard) in all three groups (Control, sdaMCI, and mdaMCI).

The percentage of correct responses (hits) and RTs (between the onset of the Go visual stimulus and pressing the key) were evaluated for each group (**Figure 2**).

**FIGURE 3:**
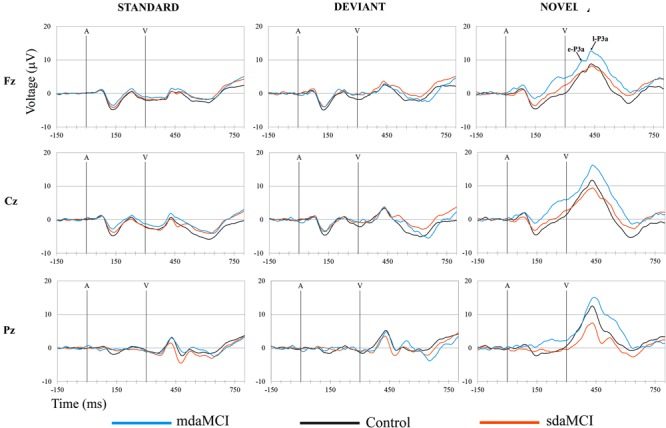
Grand-average ERP waveforms at the Fz, Cz, and Pz electrode sites, in the Standard, Deviant, and Novel conditions, comparing the three groups (Control, sdaMCI, and mdaMCI). A, auditory stimulus; V, visual stimulus. Digital bandpass filter: 0.1–20 Hz.

**FIGURE 4:**
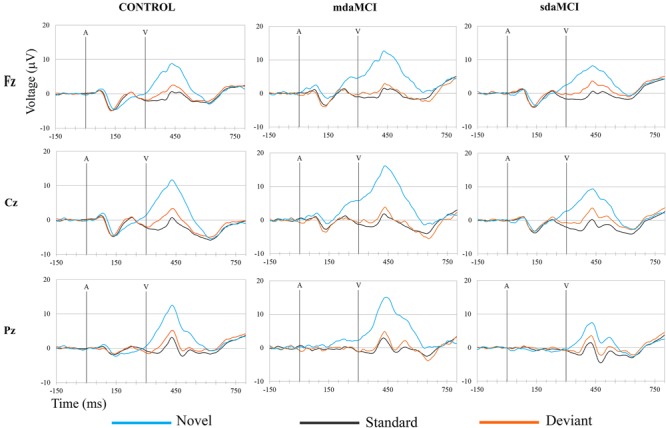
Grand-average ERP waveforms at the Fz, Cz, and Pz electrode sites, in the three groups (Control, sdaMCI, and mdaMCI), comparing the three conditions (Standard, Deviant, and Novel). A, auditory stimulus; V, visual stimulus. Digital bandpass filter: 0.1–20 Hz.

**FIGURE 5:**
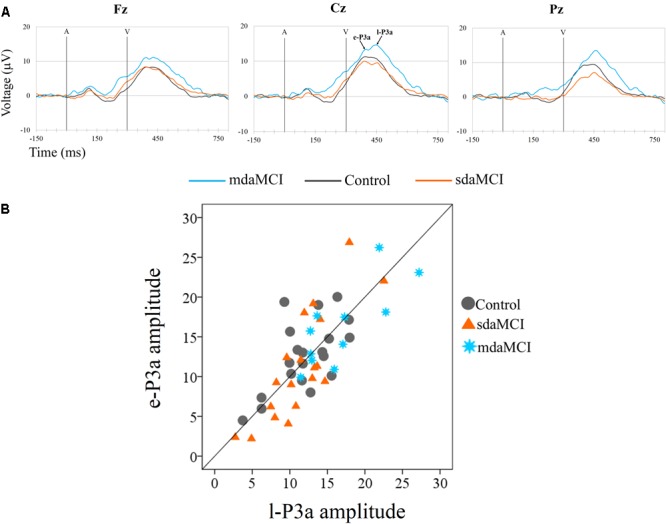
**(A)** Grand-average ERP waveforms at the Fz, Cz, and Pz electrode sites, in the Novel *minus* Standard (N-S) difference waveforms, for the three groups (Control, sdaMCI, and mdaMCI). A, auditory stimulus; V, visual stimulus. Digital bandpass filter: 0.1–20 Hz. **(B)** Scatter plot of individual dates (in the three groups) for amplitudes of e-P3a and l-P3a, at Cz electrode site, in the N-S difference waveforms.

Two phases were identified in the temporal range of P3a, for the control, sdaMCI and mdaMCI participants (see **Figures 3, 4, 5A**): (1) early P3a (e-P3a), in a latency range between 280 and 400 ms post-stimulus and with a maximum amplitude at fronto-central locations, and (2) late P3a (l-P3a), in a latency range between 350 and 500 ms post-stimulus, and with a maximum amplitude at parieto-central locations. A scatter plot of individual dates for the amplitudes of e-P3a and l-P3a (at Cz electrode site) is presented in **Figure 5B**.

Baseline-to-peak amplitudes (in microvolts) and peak latencies (in milliseconds, from the auditory stimulus onset to the maximum peak) of e-P3a and l-P3a were measured at Fz, Cz, and Pz electrode sites. Voltage and current source density (CSD) maps were obtained for topographic analysis (see **Figure 7**).

### Statistical Analysis

In order to evaluate the effects of the aMCI subtypes and the involuntary capture of attention provoked by novel stimuli, on the RT and the percentage of hits, two-factor ANOVAs with a between-subject factor *Group* (with three levels: Control, sdaMCI, and mdaMCI) and a within-subject factor *Condition* (with two levels: Standard, and Novel) were applied.

Two-factor ANOVAs (Group × Electrode Position) were applied, with the aim of evaluating the effects of sdaMCI and mdaMCI on e-P3a and l-P3a amplitudes. The *Group* factor included three levels (Control, sdaMCI, and mdaMCI) and the within-subject factor *Electrode Position* factor included three levels (Fz, Cz, and Pz).

One-factor ANOVAs (Group) were used to evaluate the effects of the aMCI subtypes on the e-P3a and l-P3a latencies at Cz, and this factor included three levels (Control, sdaMCI, and mdaMCI).

Pairwise comparison of means (with Bonferroni corrections) was carried out when the ANOVAs indicated significant results. In addition, partial eta squared ($\eta_{p}^{2}$) and Cohen’s *d* value were calculated for each significant comparison, with the aim of determining the sizes of the effects. The Cohen’s *d* analysis was carried out with G^∗^Power v.3.1.9.2 for Windows (Faul et al., 2009).

Receiver operating characteristics (ROC) curves were constructed when the Group factor exerted a main effect on the e-P3a and l-P3a parameters. As in our previous studies (Lindín et al., 2013), the ROC curve was constructed by determining the sensitivity and specificity for a range of values of the e-P3a and l-P3a parameters, and the tests were considered ideal when the area under the curve (AUC) was greater than 0.7. In addition, a line diagram was constructed with the sensitivity (true positive rate) plotted on the vertical axis and the false positive rate (1 minus specificity) on the horizontal axis.

Results were considered statistically significant at p ≤ 0.05. All statistical analyses were performed using the IBM SPSS Statistics package v.19 for Windows.

## Results

**FIGURE 6:**
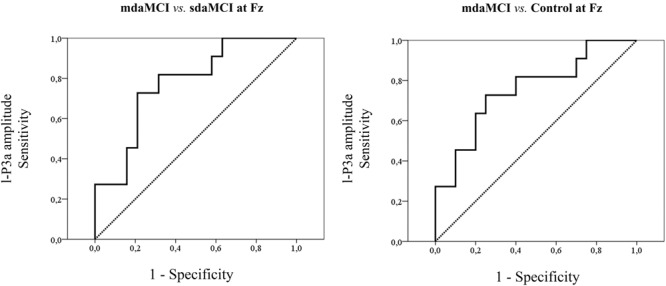
ROC curves for the amplitudes that yielded the highest sensitivity and specificity values for each type of ROC analysis.

The mean values and the standard deviations for the amplitudes and latencies of e-P3a and l-P3a components are shown in **Table 2**. The parameters of the ROC curve analysis are summarized in **Table 3**, and the ROC curves of the best markers for each type of diagnosis are presented in **Figure 6**.

**Table 2:** Mean values and standard deviations (SD, in brackets) of amplitudes (μV) and latencies (ms) for early-P3a (e-P3a, 280–400 ms) and late-P3a (l-P3a, 350-500 ms) measured in N-S difference waveforms, for the three groups of participants (Control, sdaMCI, and mdaMCI).

		Control *N* = 20	sdaMCI *N* = 19	mdaMCI *N* = 11
Amplitude e-P3a	Fz	9.5 (4.5)	9.4 (5.8)	13.3 (5.1)
	Cz	12.6 (4.4)	11.2 (6.8)	16.2 (5.1)
	Pz	10.0 (4.2)	6.6 (6.2)	12.6 (6.1)
Latency e-P3a	Cz	379 (27.6)	391 (25.0)	386 (42.7)
Amplitude l-P3a	Fz	9.2 (3.5)	9.2 (4.1)	13.1 (4.4)
	Cz	12.0 (3.9)	11.4 (4.5)	16.9 (5.1)
	Pz	10.8 (3.6)	8.8 (4.9)	15.0 (4.8)
Latency l-P3a	Cz	457 (26.1)	466 (32.1)	461 (34.5)

*sdaMCI: single-domain amnestic mild cognitive impairment; mdaMCI: multi-domain amnestic mild cognitive impairment.*

**Table 3:** Sensitivity (Se) and specificity (Sp) values and area under the curve (AUC), for the amplitudes of early-P3a (e-P3a) and late-P3a (l-P3a).

		AUC	Se	Sp
**e-P3a**	*sdaMCI vs. mdaMCI*			
	Amplitude Fz	0.72	0.73	0.74
	Amplitude Cz	0.75	0.73	0.74
	Amplitude Pz	0.79	0.91	0.68
**l-P3a**	*sdaMCI vs. mdaMCI*			
	Amplitude Fz	**0.78**	**0.73**	**0.79**
	Amplitude Cz	0.78	0.64	0.74
	Amplitude Pz	0.82	0.82	0.68
	*Control vs. mdaMCI*			
	Amplitude Fz	**0.76**	**0.73**	**0.75**
	Amplitude Cz	0.77	0.73	0.60
	Amplitude Pz	0.74	0.64	0.70

*The highest sensitivity and specificity scores for each type of ROC analysis (for those amplitudes in which the Group factor exerted a main effect) are highlighted in bold type. sdaMCI: single-domain amnestic mild cognitive impairment; mdaMCI: multi-domain amnestic mild cognitive impairment.*

### Performance

The ANOVA (Group × Condition) showed significant effects of the *Condition* factor [*F*(1,46) = 79.5; *p* < 0.001; $\eta_{p}^{2}$ = 0.64] and the *Group* × *Condition* interaction [*F*(2,46) = 5.9; *p* = 0.005; $\eta_{p}^{2}$ = 0.20] on the RT; this parameter was significantly longer (see **Figure 2**) in the Novel condition [mean (*M*) = 691 ms; standard deviation (*SD*) = 99.5] than in the Standard condition (*M* = 651 ms; *SD* = 82.7) in all three groups (*post hoc* comparison: Control: *p* < 0.001, *d* = 6.99; mdaMCI: *p* = 0.005, *d* = 7.31; sdaMCI: *p* < 0.001, *d* = 8.46).

The ANOVA (Group × Condition) showed a significant effect of the *Group* factor on the percentage of hits [*F*(1,46) = 8.7; *p* < 0.001; $\eta_{p}^{2}$ = 0.27]; this parameter was significantly higher (see **Figure 2**) in the Control (*M* = 95.6%; *SD* = 4.1) and sdaMCI (*M* = 95.0%; *SD* = 5.4) groups than in the mdaMCI (*M* = 85.9%; *SD* = 11.4) group (*post hoc* comparisons of mdaMCI *vs.* sdaMCI: *p* = 0.002, *d* = 1.17; mdaMCI *vs.* Control: *p* = 0.001, *d* = 1.2; Control *vs.* sdaMCI: *p* = 1.00, *d* = 0).

### e-P3a and l-P3a

For the e-P3a amplitude (see **Figure 5A** and **Table 2**), the ANOVA (Group × Electrode Position) revealed significant effects of the *Group* [*F*(2,47) = 3.4; *p* = 0.043; $\eta_{p}^{2}$ = 0.13] and *Electrode Position* [*F*(2,94) = 33.8; *p* < 0.001; 𝜀 = 0.7; $\eta_{p}^{2}$ = 0.42] factors, and for the *Group* × *Electrode Position* interaction [*F*(4,94) = 2.9; *p* = 0.026; $\eta_{p}^{2}$ = 0.11]. The e-P3a amplitude was significantly larger in mdaMCI than sdaMCI at Pz (*post hoc* comparisons of mdaMCI *vs.* sdaMCI: *p* = 0.017, *d* = 0.98; mdaMCI *vs.* Control: *p* = 0.637, *d* = 0.50; Control *vs.* sdaMCI: *p* = 0.172, *d* = 0.64), and close to being significantly larger in mdaMCI than sdaMCI at Cz (*p* = 0.068, *d* = 0.83). In all groups, the e-P3a amplitude was also significantly larger at the Cz than at the Fz (*p* < 0.001) and Pz (*p* < 0.001) electrode sites, and in sdaMCI group it was significantly larger at the Fz than at the Pz electrode site (*p* = 0.009).

The ROC curves for e-P3a amplitude showed sensitivity and specificity values of 0.91 and 0.68, respectively (for more details see **Table 3**) for discriminating between the mdaMCI and sdaMCI groups at the Pz electrode site (positive group: mdaMCI, negative group: sdaMCI).

For the l-P3a amplitude (see **Figure 5A** and **Table 2**), the ANOVA (Group × Electrode Position) showed significant effects of the *Group* [*F*(2,47) = 6.3; *p* = 0.004; $\eta_{p}^{2}$ = 0.21] and *Electrode Position* [*F*(2,94) = 34.0; *p* < 0.001; 𝜀 = 0.8; $\eta_{p}^{2}$ = 0.42] factors, and for the *Group* × *Electrode Position* interaction [*F*(4,94) = 2.4; *p* = 0.055; $\eta_{p}^{2}$ = 0.09].

The l-P3a amplitude was significantly larger in mdaMCI than in sdaMCI (*p* = 0.004) and Control (*p* = 0.017) groups at Fz (*post hoc* comparison of mdaMCI *vs.* sdaMCI: *p* = 0.035, *d* = 0.92; mdaMCI *vs.* Control: *p* = 0.032, *d* = 0.98; Control *vs.* sdaMCI: *p* = 1.00, *d* = 0.00), Cz (*post hoc* comparison of mdaMCI *vs.* sdaMCI: *p* = 0.006, *d* = 1.14; mdaMCI *vs.* Control: *p* = 0.014, *d* = 1.08; Control *vs.* sdaMCI: *p* = 1.00, *d* = 0.14) and Pz (*post hoc* comparison of mdaMCI *vs.* sdaMCI: *p* = 0.002, *d* = 1.28; mdaMCI *vs.* Control: *p* = 0.042, *d* = 0.99; Control *vs.* sdaMCI: *p* = 0.498, *d* = 0.47) electrode sites. Moreover, in all groups, the l-P3a amplitude was also significantly larger at the Cz than at the Fz (*p* < 0.001) and Pz (*p* < 0.001) electrode sites, and in Control group it was close to being significantly larger at Pz than at Fz (*p* = 0.060) electrode sites.

The ROC curves for l-P3a amplitude showed sensitivity and specificity values of, respectively, 0.73 and 0.79 at Fz, respectively, 0.64 and 0.74 at Cz, and, respectively, 0.82 and 0.68 at Pz for discriminating between mdaMCI and sdaMCI groups (positive group: mdaMCI, negative group: sdaMCI).

On the other hand, the ROC curves for l-P3a amplitude showed sensitivity and specificity values of, respectively, 0.73 and 0.75 at Fz, respectively, 0.73 and 0.60 at Cz, and, respectively, 0.64 and 0.70 at Pz for discriminating between mdaMCI and Control groups (positive group: mdaMCI, negative group: Control).

One-factor ANOVAs for the e-P3a and l-P3a latencies did not reveal a significant effect of the Group factor.

**FIGURE 7:**
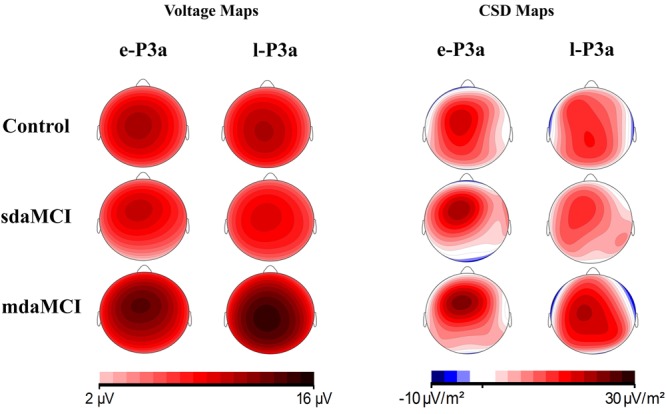
Voltage and current source density (CSD) maps for e-P3a and l-P3a maximum peaks, for the three groups (Control, sdaMCI, and mdaMCI).

Voltage maps (see **Figure 7**) for e-P3a and l-P3a also showed larger amplitudes in the mdaMCI than in the sdaMCI and Control groups. CSD maps (see **Figure 7**) revealed different sources for e-P3a and l-P3a in all three groups: a centro-frontal source was observed for e-P3a, and frontal and parietal sources (with the parietal source increased in the mdaMCI) were observed for l-P3a. These results are consistent with previous research findings in healthy adults (see Correa-Jaraba et al., 2016). In addition, frontal (in control and sdaMCI participants) and occipital (in sdaMCI and mdaMCI participants) sinks for e-P3a, and temporal sinks (in control and mdaMCI participants) for l-P3a, were observed.

## Discussion

In this ERP study, involuntary processing of irrelevant auditory stimuli followed by attended Go visual stimuli, and also RTs and hits in response to the Go visual stimuli, were evaluated in healthy adults and adults with sdaMCI and mdaMCI. The RTs were longer in the Novel than in the Standard condition in all three groups, suggesting capture of attention by novel stimuli on the performance; in addition, the percentage of hits was lower in mdaMCI participants than in the sdaMCI and control participants. The ERPs results showed significant differences between groups for both P3a phases: the e-P3a amplitude was significantly larger in mdaMCI participants than in sdaMCI participants, and the l-P3a amplitude was significantly larger in mdaMCI participants than in sdaMCI and healthy control participants. However, the e-P3a and l-P3a latencies did not differ between groups.

### Performance

The mdaMCI participants performed worse than the Control and sdaMCI participants, as reflected in the lower percentage of hits in the first group. To our knowledge, only two previous studies have evaluated differences in performance between sdaMCI and mdaMCI participants using an A-V task (Cid-Fernández et al., 2017a,b). The authors observed longer RT and fewer hits in mdaMCI participants than in control (Cid-Fernández et al., 2017a,b) and sdaMCI (Cid-Fernández et al., 2017b) participants. In the present study, our results are partly consistent with the obtained by (Cid-Fernández et al., 2017a,b), as they revealed a behavioral decline in the mdaMCI participants relative to the sdaMCI and Control participants, as reflected in a lower percentage of hits, although there were no differences in RT between groups.

On the other hand, longer RTs in response to Go visual stimuli were observed in the Novel relative to the Standard condition, suggesting a distraction effect triggered by novel stimuli in all three groups under study. This finding is consistent with those of previous studies using A-V task in healthy young adults (Escera et al., 1998, 2001; Cid-Fernández et al., 2014, 2016; Correa-Jaraba et al., 2016) and middle-aged and old adults (Cid-Fernández et al., 2014, 2016; Correa-Jaraba et al., 2016).

### e-P3a and l-P3a

In the three groups of participants, two successive phases of P3a were distinguished: an early phase (e-P3a) and a late phase (l-P3a). Both phases showed maximum amplitude at the Cz electrode site and were significantly larger at Cz relative to Fz and Pz electrode sites. In addition, a fronto-central distribution was observed for e-P3a, while l-P3a showed a parieto-central distribution, as observed in the voltage maps (**Figure 7**).

In a recent study with the same task as used in the present study, two phases of P3a were also identified in three different healthy age groups (Young, Middle-aged, and Old), in response to the novel auditory stimuli: e-P3a with a fronto-central distribution and l-P3a with a parieto-central distribution (Correa-Jaraba et al., 2016). In the aforementioned study, it was suggested that e-P3a may be a correlate of the orienting response to unexpected infrequent novel stimuli and that l-P3a may be a correlate of evaluation of these stimuli. This interpretation was based on the scalp distribution shown by the two P3a phases and on the findings of some previous studies (as cited in Correa-Jaraba et al., 2016). Specifically, differences were found for the frontal and parietal P3a (see Friedman et al., 2001), as P3a showed higher habituation at frontal than at parietal sites (Courchesne et al., 1975; Knight, 1984; Friedman and Simpson, 1994). The frontal part of P3a was considered an index of processes associated with orienting toward the stimulus (Cycowicz and Friedman, 1997; Friedman et al., 1998), because habituation is a distinctive characteristic of the orienting response (Siddle, 1991; Öhman, 1992); on the other hand, the posterior part of P3a was interpreted as probably reflecting categorization processes (Courchesne et al., 1975; Knight et al., 1995), because it showed similar features to the P3b component evoked in response to target stimuli (Friedman et al., 2001).

In the present study, both the distribution (central and parietal) and latency of l-P3a were similar to those of the P3b component. We therefore suggest that these ERP components may be identical, although further studies should be carried out to explore this possibility.

On the other hand, the findings of the present study are only partly consistent with those reported by Escera et al. (1998, 2001), who using a similar task observed two P3a consecutive phases, in response to novel auditory stimuli, in young participants: (1) an early phase, peaking between the 220 and 320 ms and with a central distribution, and (2) a late phase, with latencies about 300–400 ms and a right frontal scalp maximum. The authors proposed that the late P3a phase may reflect orienting of attention toward the irrelevant novel stimulus, because its amplitude increased when these stimuli could act as warning signals for consecutive relevant visual stimuli, relative to conditions where they did not act in this way (in a passive oddball task). On the other hand, the early P3a phase was interpreted as an index of a violation (provoked by a novel stimulus) of the regularity of an established environment model (as cited in Correa-Jaraba et al., 2016). The authors suggested that the early P3a phase may not reflect orientation toward the stimulation because “the amplitude did not increase when it acted as a warning signal, unlike in the pure passive oddball task” (as cited in Correa-Jaraba et al., 2016).

### Effects of aMCI on the e-P3a and l-P3a Parameters

Our findings revealed significant differences between the three groups of participants for the amplitude of e-P3a and l-P3a phases; however, they did not show differences between groups for the latencies.

The e-P3a amplitude was significantly larger in adults with mdaMCI than in adults with sdaMCI, which may indicate a greater involuntary capture of the attention toward the irrelevant novel stimuli in the first. Moreover, the increase in the e-P3a amplitude seems be a potential biomarker for discriminating between mdaMCI from sdaMCI participants, with good sensitivity and acceptable specificity (see **Table 3**).

The l-P3a amplitude was also significantly larger in the mdaMCI groups than in sdaMCI and Control groups, which may indicate that participants with mdaMCI allocate more attentional resources for evaluating irrelevant novel stimuli. The increase in the l-P3a amplitude also seems to be a potential biomarker for mdaMCI, as it was able to discriminate between the mdaMCI and Control groups, and mdaMCI and sdaMCI groups, with moderate sensitivity and specificity (see **Table 3**).

Further studies should be carried out with larger samples of participants to confirm our results and also to evaluate the clinical usefulness of changes in e-P3a and l-P3a amplitudes for identifying aMCI subtypes.

The observed differences between the mdaMCI group and the other two groups (healthy controls and sdaMCI), together with the absence of significant differences between the sdaMCI and Control groups, provide evidence for the need to distinguish aMCI subtypes with the aim of evaluating the P3a component as a neurocognitive marker for diagnosing aMCI. Thus, it is possible that the similarity between the P3a amplitudes of sdaMCI and Controls may mask the differences between mdaMCI and Controls.

The need to distinguish the aMCI subtypes was also evidenced in our previous ERP studies in which other (Cespón et al., 2015) or similar (Cid-Fernández et al., 2017a,b) cognitive tasks were used. These studies revealed behavioral and neurocognitive decline in mdaMCI participants relative to the sdaMCI and control participants. Specifically, a decrease in the allocation of attentional resources to target stimuli was observed in mdaMCI participants relative to healthy controls, with no difference between controls and sdaMCI participants (Cespón et al., 2015), as well as deficits in executive processes in mdaMCI relative to sdaMCI and control adults (Cespón et al., 2015; Cid-Fernández et al., 2017a,b). Studies using neuroimaging techniques or histopathologic analysis also supported the need to differentiate the two aMCI subtypes, as adults with mdaMCI showed different brain damage than those with sdaMCI, e.g., a more widespread white matter degeneration (Li et al., 2013; Liu et al., 2017) and higher β-amyloid (Aβ) deposition (for a review see Ong et al., 2013).

Several follow-up studies showed that the risk of conversion to AD is higher in mdaMCI than in sdaMCI (Tabert et al., 2006; Caffarra et al., 2008). In the present study, the larger e-P3a and l-P3a amplitudes shown by the mdaMCI group than by the sdaMCI group may also be optimal markers of possible progression to AD; however, this assumption would be better addressed in a longitudinal study comparing aMCI subtypes.

Cecchi et al. (2015) obtained smaller P3a amplitude in adults with mild AD than in healthy controls; however, other authors found no difference in this parameter between both groups (Yamaguchi et al., 2000). Given that patients with AD showed changes in P3a parameters relative to controls, we expected to find similar changes in aMCI, especially in the mdaMCI group. In this respect, the results of the present study are partly consistent with the findings for AD, because we observed significant differences between the mdaMCI group and the sdaMCI and/or Control groups, with significantly larger e-P3a and l-P3a amplitudes in the mdaMCI group. In addition, we did not observe any differences between the sdaMCI and Control groups in e-P3a or l-P3a parameters. First, it should be noted that reports of the effects of AD, and other types of dementia, on P3a are scarce, and the findings are to some extent inconsistent. Moreover, due to the lack of research on the P3a component in people with aMCI, comparison of our findings with other relative findings is difficult. The discrepancies between the results of the present and previous studies with AD patients may stem from variations in the methods used, e.g., related to different task characteristics.

In summary, healthy, sdaMCI, and mdaMCI participants performed an auditory-visual distraction-attention task while EEG activity was recorded. A greater orienting response to unattended novel auditory stimuli, and allocation of more attentional resources for the subsequent evaluation of these stimuli (as indicated by larger e-P3a and l-P3a amplitudes, respectively) were observed in the mdaMCI group relative to the sdaMCI and Control groups. In addition, the sensitivity and specificity scores provide evidence supporting the use of larger e-P3a and l-P3a amplitudes as neurocognitive markers of mdaMCI in the clinical setting. Follow-up studies should investigate the predictive utility of e-P3a and l-P3a amplitudes to identify people with aMCI who will develop AD and those who will remain stable.

## Author Contributions

KC-J: electroencephalographic recordings and event-related potential processing, interpretation of data, and drafting the manuscript. ML and FD: study design, monitoring of procedure and methodology, data interpretation and critical review of the manuscript for important intellectual content, and final approval of the version for publication.

## Conflict of Interest Statement

The authors declare that the research was conducted in the absence of any commercial or financial relationships that could be construed as a potential conflict of interest.
